# Upgrading of Automotive Polymer Fractions Obtained by a Flotation Separation Process: Properties and Structure

**DOI:** 10.3390/polym18151917

**Published:** 2026-08-05

**Authors:** Wiktoria Kanciak, Dorota Czarnecka-Komorowska, Mikołaj Popławski

**Affiliations:** 1 Institute of Materials Technology, Poznan University of Technology, Piotrowo 3, 61-138 Poznan, Poland; dorota.czarnecka-komorowska@put.poznan.pl; 2 Institute of Materials Engineering, Poznan University of Technology, Jana Pawła II 24, 60-965 Poznan, Poland; mikolaj.poplawski@put.poznan.pl

**Keywords:** circular economy, plastics recycling, automotive industry, flotation separation, compatibilisation of polymer mixtures

## Abstract

This study investigated the upgrading process of polymer fractions obtained from automotive waste by a three-stage flotation–sedimentation separation process. The separated automotive recycled blend (ARB) fraction was modified with a compatibiliser at contents of 5, 10 and 15 wt%, and the resulting blends were processed by extrusion and injection moulding. Multipoint FTIR analysis confirmed the multicomponent character of the ARB fraction and indicated the presence of, among others, polycarbonate (PC), acrylonitrile-butadiene-styrene (ABS), high-density polyethylene (HDPE) and polystyrene (PS). The influence of compatibiliser addition on density, Shore D hardness, tensile properties, thermal behaviour and fracture-surface morphology was evaluated using gas pycnometry, static tensile testing, differential scanning calorimetry (DSC) and scanning electron microscopy (SEM). The results showed that increasing compatibiliser content gradually reduced the density of the blends, while hardness remained at a level comparable to that of the reference material. Tensile testing indicated that the addition of the compatibiliser reduced Young’s modulus, whereas ultimate tensile strength and tensile stress at break were maintained close to the unmodified blend. Among the modified blends, the most favourable balance of the evaluated properties was observed for the ARB/10C sample containing 10 wt% compatibiliser. This blend exhibited the highest tensile stress at break among the investigated blends and a comparatively compact fracture-surface morphology. DSC analysis showed similar principal thermal transition ranges for all blends. The results indicate that 10 wt% compatibiliser is an optimal addition for upgrading recycled automotive polymer fractions.

## 1. Introduction

Contemporary industrial development is increasingly oriented towards reducing negative environmental impacts, ensuring the rational management of resources and decreasing the amount of waste generated at different stages of the product life cycle [1,2,3]. These assumptions are consistent with the concept of sustainable development, which aims to reconcile economic progress with environmental protection and the responsible use of raw materials [1,4,5]. In this context, the circular economy is gaining particular importance, as it assumes a departure from the traditional linear model based on the sequence of production, use and waste disposal [1,2,4,5]. By contrast, the circular model focuses on extending the service life of materials and products, reducing the consumption of primary raw materials and maximising the reuse of waste as valuable secondary raw materials [1,2,6]. In the case of plastics, these measures are particularly important due to the large scale of their production, their widespread use and the problems associated with the management of plastic waste [3,6,7,8,9].

Polymeric materials play an important role in many industrial sectors, including the automotive industry, where they are used due to their low weight, favourable mechanical properties, corrosion resistance and considerable design freedom in the manufacture of components with complex geometries. Their application enables a reduction in vehicle weight, which may contribute to lower energy consumption during operation [10,11,12]. At the same time, the increasing share of polymeric materials in vehicle construction leads to a growing amount of plastic waste generated after the end of a vehicle’s service life [12,13,14,15]. However, the recycling of these materials is hindered by their heterogeneous composition, the presence of additives, fillers, coatings, adhesives and their combination with other materials [12,13,14,15,16]. For this reason, the management of polymer waste from the automotive sector requires the use of appropriate methods for material preparation, separation and modification, enabling the recovery of fractions suitable for reprocessing [13,14,15,16,17]. The development of such solutions constitutes an important element in the implementation of circular economy principles in the automotive sector and supports the reduction in demand for primary plastic raw materials [13,14,15,17].

In the case of post-consumer automotive components, such as vehicle headlamps, their complex material composition and construction, resulting from the need to meet demanding performance requirements, constitute a particular challenge [12,18,19]. These components are characterised by adequate mechanical resistance, dimensional stability, resistance to weathering and, in the case of optical parts, good light transmittance [18,19]. For this reason, various plastics are used in their manufacture, including polycarbonate, polymethyl methacrylate and polypropylene, as well as additional metallic elements, seals, protective coatings, adhesives and decorative layers [12,18,19]. Such material diversity means that headlamps, after the end of a vehicle’s service life, cannot be treated as a homogeneous material stream, and their recycling requires an appropriately planned preparation process [12,18,19].

The management of headlamps from passenger cars is challenging not only because of the presence of many different material types, but also because the plastics originating from headlamps may differ, among other factors, in molecular weight, additive composition, colour and surface properties [8,9,12,18]. In practice, this means that waste size reduction alone is not sufficient to obtain a material with stable properties [7,8,9,20]. It is therefore necessary to apply further stages, such as cleaning, sorting and separation of material fractions, which make it possible to increase the homogeneity of the secondary raw material and prepare it for subsequent reprocessing [20,21,22].

One of the methods used for the separation of polymer mixtures is density-based sink–float separation, also referred to as flotation–sedimentation or sinking–flotation separation [22,23,24,25,26]. The method is based on differences between the densities of the polymer particles and the separation medium. Particles with a density lower than that of the liquid remain in the floating fraction, whereas particles with a higher density sediment. By adjusting the density of the separation medium in consecutive stages, polymer fractions within selected density ranges can be progressively isolated [22,23,24,26].

Water is commonly used as the first separation medium because it enables low-density polymers, particularly polyethylene and polypropylene, to be separated from polymers with densities exceeding approx. 1 g cm^−3^. In subsequent stages, aqueous salt solutions, alcohol solutions, or other density-modifying media may be used to obtain fractions with narrower density ranges [22,23,24,26]. The efficiency of the process is affected by the density and stability of the medium, particle size and shape, separation time, agitation conditions, the degree of material liberation and the presence of contaminants. Fillers, pigments, coatings, adhered impurities and trapped air may modify the apparent density of waste particles and thereby influence their behaviour in the separating liquid [22,23,25,26].

Density-based sink–float separation should be clearly distinguished from froth flotation. In sink–float separation, the decisive factor is the relationship between the density of the particle and that of the liquid medium. Froth flotation, in contrast, is governed mainly by differences in surface wettability and the selective attachment of particles to air bubbles [27,28]. Nevertheless, froth flotation may be used as an additional purification stage when polymers have similar or overlapping densities, as demonstrated in hybrid separation systems combining sink–float separation with froth flotation [24,29].

The polymer fractions obtained as a result of sink–float separation, despite being partially organised in terms of density, may still exhibit the characteristics of multicomponent mixtures [23,24,25,26,29]. The presence of different polymer phases within a single material may lead to limited compatibility between the components, poor interfacial adhesion and structural heterogeneity [30,31,32]. In practice, this results in processing difficulties and the possible deterioration of the functional properties of the obtained materials [7,8,30,31]. For this reason, the isolation of fractions within a defined density range alone is not always sufficient to obtain a secondary raw material with properties that allow its reuse in technical products [30,31,32].

One method for improving the quality of recycled polymer mixtures is the use of compatibilisers. These additives are intended to improve the interactions between the components of the mixture, reduce phase separation and increase the structural homogeneity of the material. This may promote a more uniform transfer of stresses within the material, stabilise mechanical properties and improve the reproducibility of processing parameters [30,31,32]. In the case of recyclates originating from the automotive industry, this is particularly important, as materials intended for reuse should exhibit an appropriate level of strength, thermal stability and predictable behaviour during processing [12,14,15,30].

Compatibilisation may proceed through non-reactive or reactive mechanisms. Non-reactive compatibilisers, including selected block and graft copolymers, are preferentially located at the interface between immiscible polymer phases and reduce interfacial tension. Reactive compatibilisers contain functional groups capable of reacting with one or more blend components during melt processing, leading to the in situ formation of interfacial copolymers [30,31,32]. In both cases, improved interfacial adhesion and reduced coalescence of the dispersed phase can produce a finer and more stable morphology and facilitate stress transfer between the phases. However, compatibiliser efficiency depends on the chemical composition and proportions of the polymer phases, the molecular architecture and concentration of the additive, the viscosity ratio of the components and the applied processing conditions [30,31,32,33].

Assessing the effect of a compatibiliser on the properties of separated polymer fractions makes it possible to determine whether this type of modification may constitute an effective route for their further management [30,31,32]. This is significant from both technological and environmental perspectives, as it enables an increase in the functional value of waste-derived materials and supports their reintroduction into the material cycle [1,2,14,15,30].

The literature increasingly emphasises the need to develop methods enabling the effective recycling of plastics originating from end-of-life vehicles. Gall et al. [15] compared polymer recyclates derived from end-of-life vehicles with packaging-derived recyclates, indicating that materials from the automotive sector are still returned to the production of new products only to a limited extent, despite the growing requirements concerning the share of secondary raw materials in vehicles. The authors noted that increasing the use of recyclates in the automotive industry requires not only effective material recovery but also a detailed assessment of the properties of secondary raw materials [15].

Wang et al. [27] presented a review of flotation separation methods for plastic waste. The authors discussed the specific characteristics of polymer froth flotation and indicated that this method may be an effective supplementary tool for separating plastic mixtures with different surface properties. They also emphasised the importance of appropriately selecting process conditions, including the modification of polymer surface wettability, in order to obtain fractions with higher material purity [27].

Meneses Quelal et al. [23] investigated the possibility of separating selected polymers and post-consumer plastic waste using the sinking–flotation method. Various separating media were used in the study, including water, ethanol solutions and sodium chloride solutions of different densities. The results confirmed that the selection of the separating medium is of key importance for the efficiency of polymer mixture separation, which is significant in the context of preparing material fractions for further processing [23].

A similar issue was addressed by Pita [29], who analysed the separation of a mixture of six post-consumer plastics using a combination of sink–float separation and froth flotation. The author demonstrated that the application of several separation stages enables more effective separation of complex polymer mixtures, particularly when individual plastics have similar properties or occur in the waste stream as a multicomponent system. These findings indicate the validity of using multistage separation processes in plastics recycling [29].

Pongstabodee et al. [24] investigated a three-stage sink–float process combined with selective flotation for a mixture of post-consumer plastics. Their results demonstrated that sequential changes in the density of the separation medium enabled the preliminary isolation of several polymer groups from a complex waste stream. Van den Eynde et al. [25] evaluated density-based sorting of plastics originating from waste electrical and electronic equipment and end-of-life vehicles and showed that its effectiveness may be limited by overlapping polymer densities and additives that alter material density. Fiorente et al. [26] confirmed that the type and concentration of the separating medium strongly affect the recovery and purity of polymer fractions obtained from a real waste stream.

Another important research direction is the compatibilisation of recycled polymer mixtures. In their review on the mechanical recycling of mixed thermoplastic waste, Maris et al. [30] indicated that compatibilisers can improve interfacial interactions, reduce phase separation and stabilise the morphology of polymer mixtures. The authors emphasised that appropriately selected compatibilisation is one of the key tools for increasing the potential reuse of multicomponent materials [30].

Dorigato [31] further emphasised that the properties of recycled polymer blends are strongly related to their phase morphology, processing history and the effectiveness of interfacial modification. Self et al. [32] described block, graft and multiblock copolymers as advanced compatibilisers capable of stabilising interfaces between immiscible polymers. Muzata et al. [33] noted that the compatibilisation of real post-consumer recyclates is more complex than the modification of model binary blends because actual waste streams may contain polymers of different grades, degradation products, fillers, pigments and residual contaminants. In a PP/PC model system relevant to automotive headlamp waste, Jasinska-Walc et al. [34] showed that appropriately designed block and graft copolymers promoted the formation of a finer, more stable phase morphology and improved the melt strength of compatibilised blends.

The aim of this study was to assess the possibility of managing a polymer fraction obtained through a three-stage flotation–sedimentation separation process of materials originating from the automotive industry. In the subsequent stage, the separated fraction was upgraded by incorporating a compatibiliser into the mixture. The resulting mixtures were processed by extrusion and injection moulding, after which their physicomechanical properties and fracture-surface morphology were evaluated. The results obtained made it possible to determine the effect of compatibilisation on the behaviour of the investigated materials and to indicate a potential route for their reuse in accordance with the principles of the circular economy.

## 2. Materials and Methods

### 2.1. Materials

**Figure 1:**
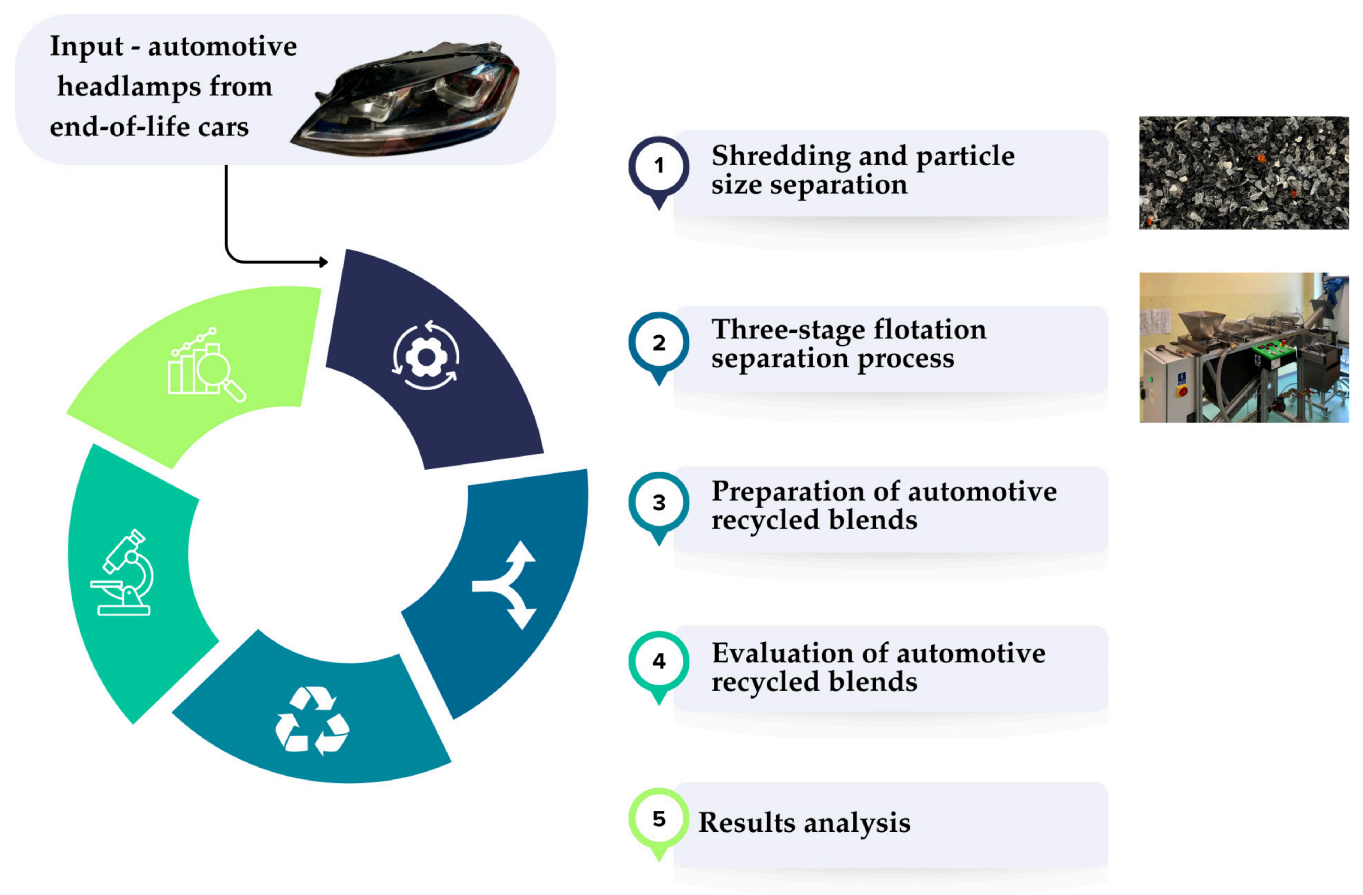
Diagram of the conducted work.

The subject of the study was separated polymer fractions originating from post-consumer passenger car headlamps recovered from the dismantling of end-of-life vehicles in accordance with the principles set out in the End-of-Life Vehicles (ELV) Act. A diagram of the conducted work is presented in Figure 1.

In the first stage, the headlamps were size-reduced using a Shini SG-1411-CE-X knife mill (Shini Plastics Technologies Inc., New Taipei City, Taiwan). The plastics were then separated according to particle size by sieve analysis using a Vibratory Sieve Shaker Fritsch ANALYSETTE 3 120 Pro apparatus (FRITSCH GmbH, Idar-Oberstein, Germany). Sieves with mesh sizes ranging from 0.8 to 4 mm were used in the analysis. The measurement time was 5 min, with a 5 s interval, while the amplitude was set at 1 mm.

After completion of the flotation–sedimentation process, sieve analysis was repeated for the recovered fraction subsequently used for blend preparation. The analysis was conducted according to the same procedure as that applied to the feed material in order to evaluate possible changes in particle-size distribution during separation.

### 2.2. Separation Process

**Figure 2:**
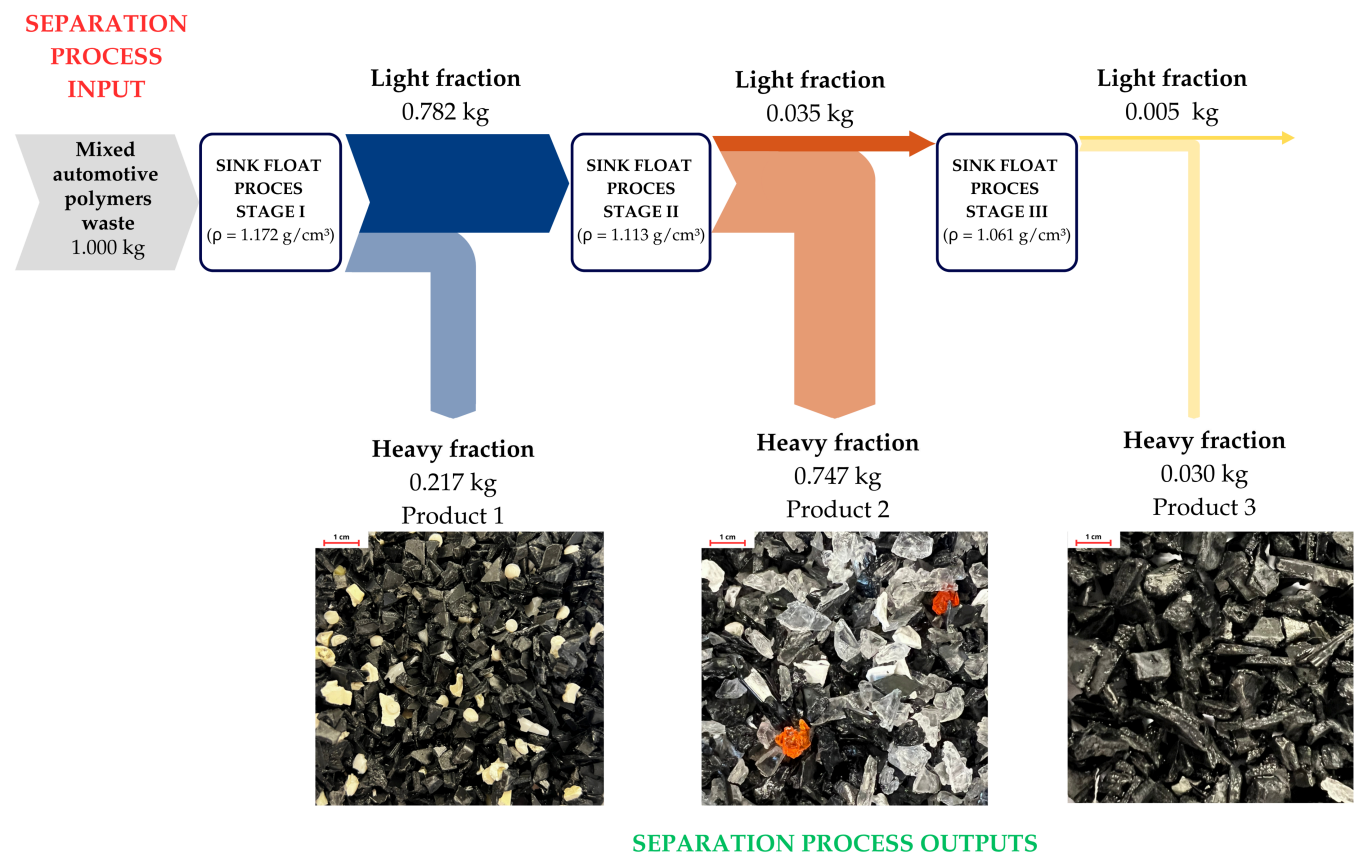
Diagram of the three-stage flotation separation process.

The separation process was carried out in several stages according to the diagram presented in Figure 2.

**Figure 3:**
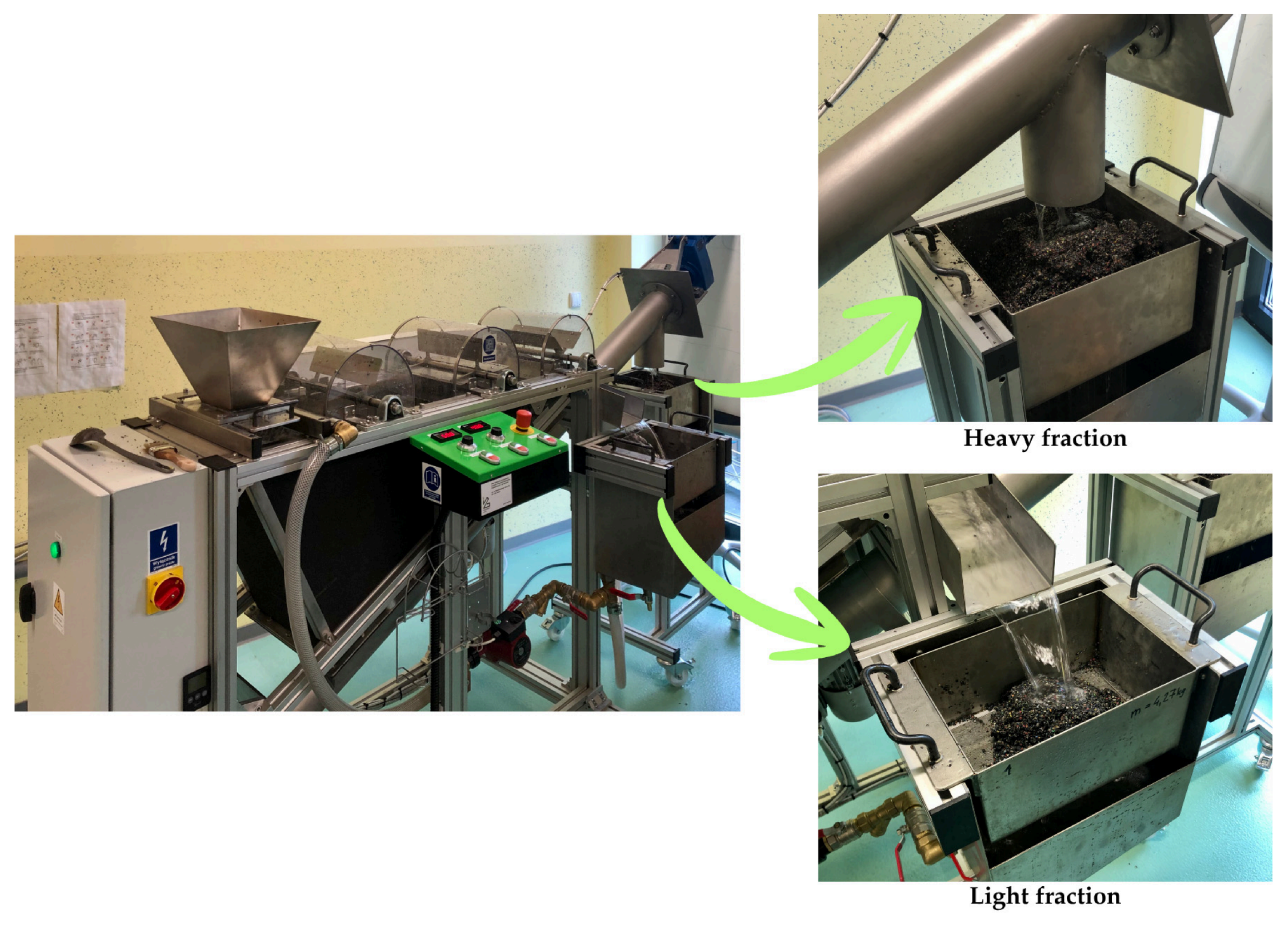
Flotation tank for plastics (Poznan University of Technology, Poznan, Poland).

The separation was performed using a flotation tank for plastics (Figure 3) under the following process parameters: the screw speed was 50 rpm, the paddle speed was 40 rpm and the temperature of the flotation medium was 20 °C.

The process was conducted as a function of density. For this purpose, three potassium hydroxide solutions with defined specific densities were prepared, which enabled the flotation separation parameter to be varied. The prepared solutions included a 25% solution with a density of 1.172 g/cm^3^, a 17% solution with a density of 1.113 g/cm^3^ and an 11% solution with a density of 1.061 g/cm^3^. The density of the obtained solutions was measured using a Densito density meter (Mettler Toledo, Greifensee, Switzerland).

The light stream obtained during each of the first two stages was used as the feed material for the subsequent separation stage. The light and heavy streams obtained after each stage were collected and weighed. Stage mass yields and mass-balance closure were calculated relative to the feed mass introduced into the corresponding stage.

The fraction operationally recovered as the heavy stream at a separation-medium density of 1.113 g/cm^3^, after removal of the fraction denser than 1.172 g/cm^3^, was selected for further investigation and designated as the automotive recycled blend (ARB). The composition and designations of the mixture subjected to compatibilisation are presented in Table 1. The ARB blend was modified using the ENGAGE compatibiliser from Dow, (Dow Inc., Midland, MI, USA) [35]. It is an ethylene-octene polyolefin elastomeric modifier with a potential compatibilising effect. According to the manufacturer’s technical data sheet, the material has a density of 0.875 g/cm^3^ and a melt index of 3.0 g/10 min at 190 °C/2.16 kg. Its reported glass-transition, melting temperature and peak crystallisation temperature are approx. −51 °C, 66 °C and 48 °C, respectively. Its application was aimed at improving the interactions between the components of the blend and increasing the homogeneity of the obtained material. A compatibiliser was incorporated into the automotive recycled blend (ARB) at contents of 5, 10 and 15 wt%, resulting in the ARB/5C, ARB/10C and ARB/15C blends, respectively by mixing in a twin-screw extruder.

The materials were processed using a twin-screw extruder (ZAMAK Mercator, Skawina, Poland) in order to homogenise the composition and prepare the regranulate. Standardised test specimens were produced by injection moulding from the prepared regranulate. The process was carried out at barrel heating zone temperature settings of 250, 250, 230 and 220 °C, respectively, with a mould temperature of 50 °C. The shot volume was 12.00 cm^3^, and the injection speed was 20.0 cm^3^/s. The holding pressure time was 5 s, and the cooling time was set at 10 s. The process parameters were selected to ensure proper plasticisation of the material, complete filling of the mould cavity and repeatability of the obtained specimens. The resulting blends were then evaluated in terms of selected physical and mechanical properties and subjected to microscopic observation.

### 2.3. Research Methods

Material identification of the selected fractions was carried out using Fourier-Transform Infrared Spectroscopy (FTIR). Measurements were performed using a JASCO FT/IR-4600 (Tokyo, Japan) type A spectrometer. Five independently selected locations were analysed for each material in order to account for the heterogeneous character of the recycled blends. Polymer identification was based on combinations of characteristic absorption bands and comparisons with reference spectra available in spectral libraries.

The mechanical properties of the produced polymer blends were evaluated by static tensile testing in accordance with PN-EN ISO 527-2 [36]. The measurements were performed at room temperature using a Zwick Roell Z010 universal testing machine (ZwickRoell GmbH & Co. KG, Ulm, Germany) at a crosshead speed of 50 mm/min. Five specimens were tested for each blend. Young’s modulus, tensile strength and tensile stress at break were determined, and the results are reported as mean values with the corresponding standard deviations.

The hardness of the investigated materials was determined using the Shore D method with a Sauter durometer (SAUTER GmbH, Balingen, Germany), in accordance with the PN-EN ISO 868 standard [37]. Measurements were carried out at room temperature on the surface of the prepared test specimens. A series of 10 measurements were performed for each blend, and the results are reported as mean values with the corresponding standard deviations.

Statistical analysis was performed using Welch’s one-way ANOVA (OriginPro 2026) followed by the Games–Howell post hoc test. Differences were considered statistically significant at *p* < 0.05.

The density of the samples was determined by gas pycnometry using an Anton Paar Ultrapyc 5000 instrument (Anton Paar QuantaTec Inc., Boynton Beach, FL, USA). in accordance with ISO 1183-3 [38]. Nitrogen was used as the analysis gas, and the target pressure was set to 18.0 psi. All measurements were performed at a controlled temperature of 20.0 °C using a 10 cm^3^ measurement cell. Samples of known mass were placed in the measurement cell and subjected to a 0.3 min gas-flow cycle before analysis. The true volume of each sample was determined from the pressure changes recorded after the introduction of the analysis gas, and the density was calculated from the measured mass and volume. For each blend, ten measurements were performed, and the results are reported as mean values with the corresponding standard deviations. Gas pycnometry enables the accurate measurement of materials with irregular shapes and is particularly suitable for heterogeneous polymer blends and recyclates [39].

Differential scanning calorimetry (DSC) was performed using a NETZSCH DSC 204 F1 Phoenix (NETZSCH-Gerätebau GmbH, Selb, Germany) apparatus to analyse the thermal properties of the investigated blends. The test was carried out in accordance with PN-EN ISO 11357-1 [40], under a nitrogen atmosphere. The samples were subjected to two complete heating and cooling cycles over a temperature range from 20 to 250 °C at a rate of 10 K/min. The obtained DSC curves were used to evaluate the thermal transitions occurring in the materials, including the determination of characteristic temperatures and changes associated with the presence of different polymer fractions and the addition of the compatibiliser.

In order to analyse the fracture behaviour, a microscopic assessment of the fracture surfaces was carried out. The observations were performed using a Tescan Mira 3 scanning electron microscope (SEM) (TESCAN GROUP, a.s., Brno, Czech Republic). The observations were performed using the secondary-electron detector at an accelerating voltage of 12.0 kV. The SEM examination was used as a complementary method for comparing fracture-surface continuity, heterogeneity and visible discontinuities among the investigated materials. The obtained microscopic images complemented the results of the physical and mechanical tests, allowing the material properties to be correlated with their structure.

## 3. Results

### 3.1. Particle-Size Distribution and Separation Results

**Figure 4:**
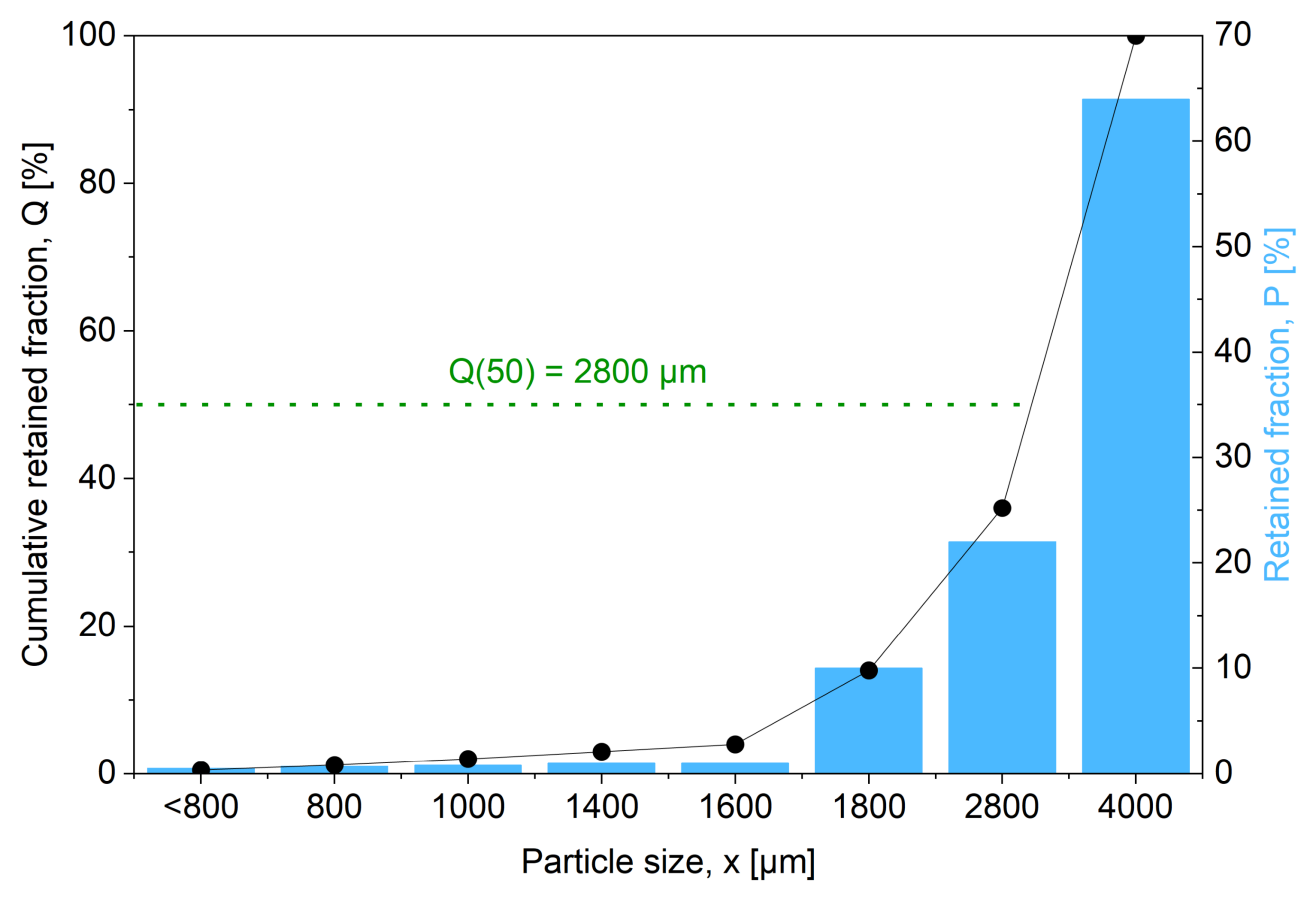
Cumulative particle size distribution curve (Q) and recalculated oversize fraction (P) of the investigated plastic mixture.

The results of the sieve analysis are presented in Figure 4.

Figure 4 presents the recalculated oversize fraction (P) as a histogram, while the cumulative particle-size distribution curve (Q) is shown as a line plot. The largest mass fraction corresponded to the 4000 μm size class and accounted for approximately 65% of the total sample mass. The second-largest fraction corresponded to 2800 μm and accounted for approximately 22%, whereas the remaining fractions each represented less than 10%. The cumulative distribution curve indicates a median particle size, Q(50), of approximately 2800 μm, meaning that 50% of the sample mass consisted of particles with sizes equal to or smaller than 2800 μm.

Sieve analysis repeated for the ARB fraction recovered after flotation–sedimentation separation showed a particle-size distribution comparable to that of the feed material. No substantial redistribution of particle sizes was observed. Therefore, the recovered ARB fraction was used in the subsequent processing and characterisation stages.

The mass balance and stage yields of the three-stage separation process are presented in Table 2.

In the first stage, 1.000 kg of feed material produced 0.782 kg of the light fraction stream and 0.218 kg of the heavy fraction stream. The light fraction stream obtained during the first stage was introduced into the second stage, in which 0.747 kg of the heavy fraction stream and 0.035 kg of the light fraction stream were recovered. In the third stage, the remaining 0.035 kg of the light fraction stream produced 0.030 kg of the heavy fraction stream and 0.005 kg of the light stream.

The fraction selected for further processing, designated as ARB, was recovered as the heavy product during the second separation stage and represented 74.7 wt% of the initial feed mass. The applied sequence of separation media enabled the operational recovery of this fraction within the density window defined by separation-medium densities of 1.113 and 1.172 g/cm^3^.

### 3.2. FTIR Identification

**Figure 5:**
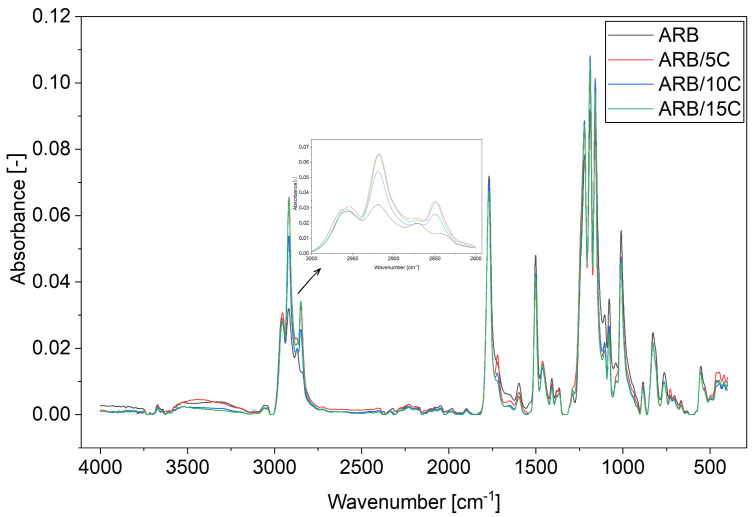
FTIR spectra of analysed materials.

Figure 5 presents the FTIR spectra of the ARB material and the blends containing 5, 10 and 15 wt% compatibiliser. The spectra of all investigated blends showed a generally similar band pattern, and no additional distinct absorption bands were observed after modification.

The qualitative identification of the polymer components was based on characteristic absorption bands observed over the full spectral range and comparison with reference library spectra. The carbonyl band near 1770 cm^−1^, together with aromatic and -C–O–C absorption bands, supported the identification of polycarbonate. The nitrile absorption band in the region of approximately 2230–2240 cm^−1^, accompanied by styrenic aromatic bands, indicated the presence of ABS. Bands characteristic of aliphatic -CH_2_ groups, including those near 2920 and 2850 cm^−1^, supported the identification of HDPE-containing fractions. Aromatic bands in the regions of approximately 1600–1500 and 760–700 cm^−1^ were associated with styrenic components, including PS. The analysis therefore indicated that the ARB blend contained, among others, PC, ABS, HDPE and PS.

The enlarged region between 3000 and 2800 cm^−1^ shows the aliphatic -C–H stretching bands. The bands near 2955, 2920 and 2850 cm^−1^ are assigned to asymmetric -CH_3_ stretching and asymmetric and symmetric -CH_2_ stretching vibrations, respectively. Differences in their intensities are consistent with changes in the contribution of aliphatic hydrocarbon segments after the addition of the ethylene-octene modifier.

### 3.3. Density

**Figure 6:**
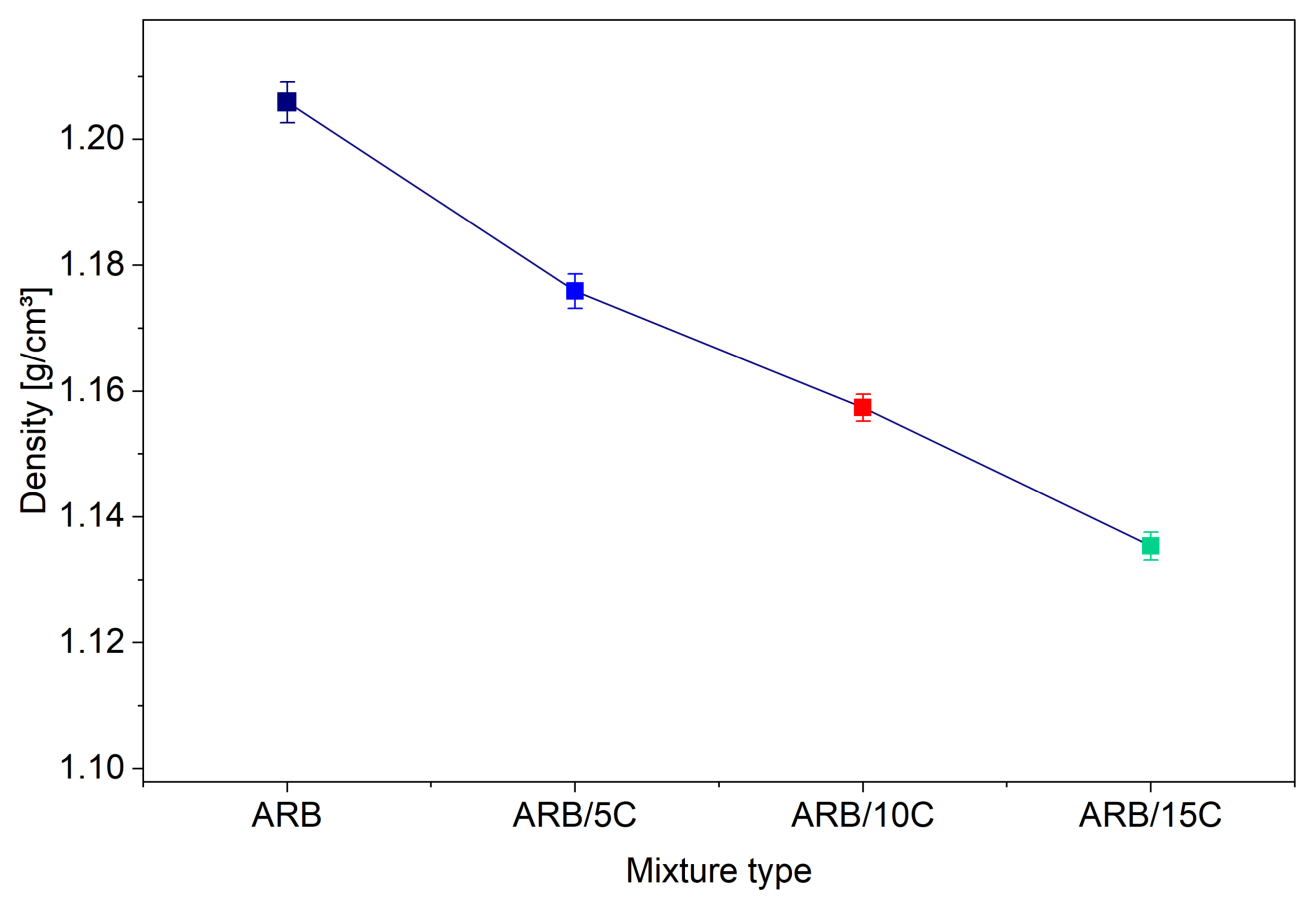
Density of the investigated polymer blends as a function of compatibiliser content. The symbol represents the mean density value with the corresponding standard deviation for each material, n = 10.

Figure 6 presents a graph illustrating the effect of compatibiliser addition on the density of the investigated blends.

The ARB blend without compatibiliser showed the highest density, amounting to approximately 1.20 g/cm^3^. The incorporation of the compatibiliser resulted in a systematic decrease in density with increasing content of this additive in the composition. For the material containing 5% compatibiliser, the density decreased to approximately 1.18 g/cm^3^, whereas at a 10% addition it reached approximately 1.16 g/cm^3^. The lowest density was recorded for the sample containing 15% compatibiliser, for which the value was approximately 1.14 g/cm^3^.

The observed trend indicates that increasing the compatibiliser content leads to a gradual decrease in the density of the investigated material system. This may primarily result from the lower density of the compatibiliser itself, approximately 0.875 g/cm^3^ [35], compared with the density of the base material. The incorporation of a lower-density component into the polymer blend may reduce the average density of the entire system. In addition, these changes may be associated with modifications in the internal structure of the material, including changes in the packing of polymer phases, a reduction in the degree of matrix compaction, or the formation of a larger free volume within the material. The decrease in density may also suggest changes in interfacial interactions occurring after the incorporation of the compatibiliser.

### 3.4. Mechanical Properties

**Figure 7:**
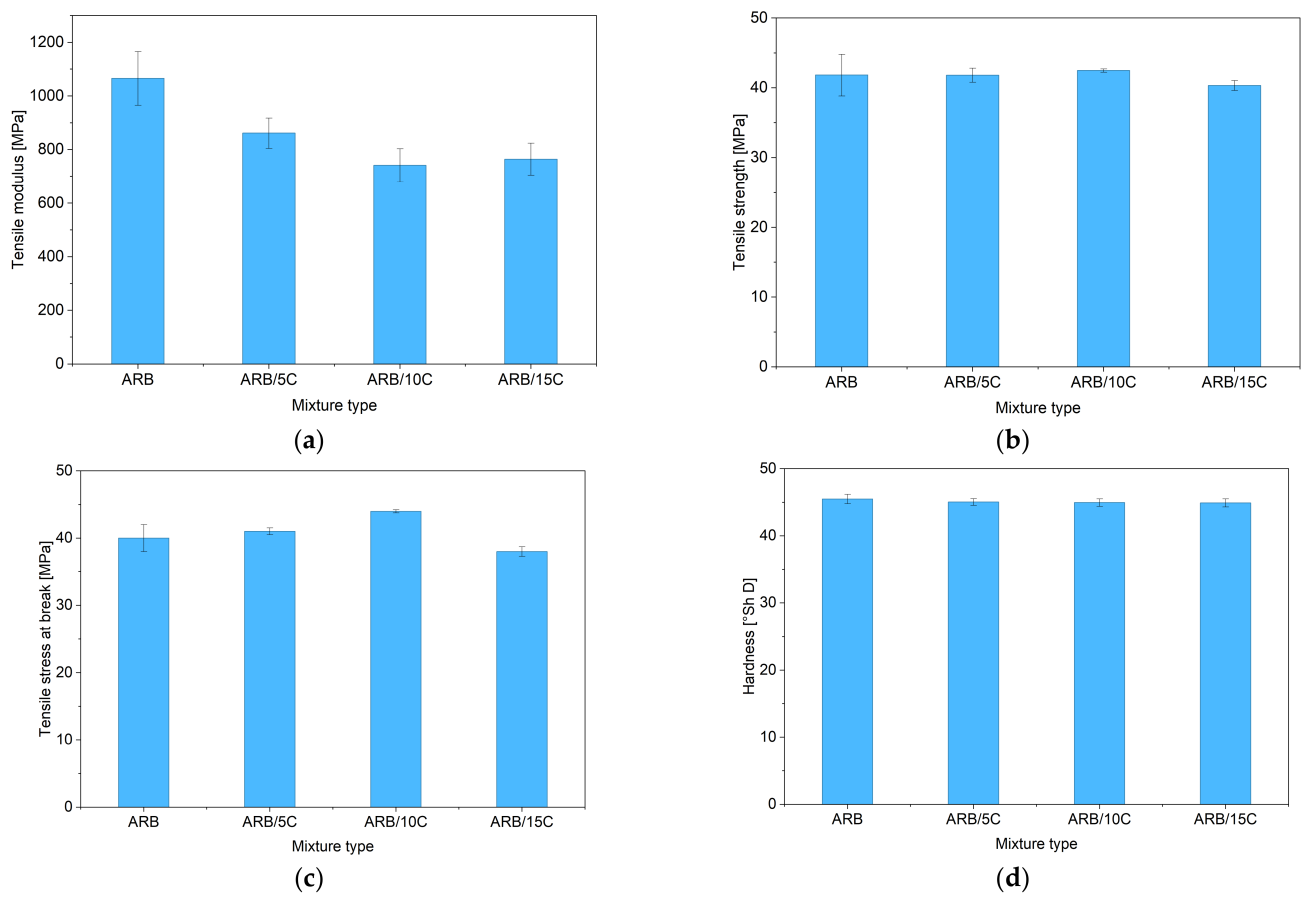
Mechanical properties of ARB blends: (**a**) Young’s modulus, (**b**) tensile strength, (**c**) tensile stress at break and (**d**) Shore D hardness.

The mechanical properties were evaluated based on the static tensile test results (Figure 7a–c) and hardness measurements (Figure 7d), as well as the data summarized in Table 3.

In the case of Young’s modulus, presented in Figure 7a, the highest value was obtained for the reference material ARB, which did not contain the compatibiliser. The introduction of the additive resulted in a reduction in material stiffness. For the ARB/5C sample, the modulus decreased by approximately 19% compared with ARB, whereas for the ARB/10C sample the decrease was approximately 31%. In the case of the ARB/15C blend, a slight increase in modulus was observed compared with the ARB/10C sample; however, this value still remained lower than that obtained for the reference material, by approximately 28%. The observed trend may indicate that the compatibiliser affects the structure of the blend and partially reduces its stiffness, increasing the susceptibility of the material to deformation.

The tensile strength, presented in Figure 7b, remained at a similar level for all the analysed materials. Compared with the reference sample ARB, the addition of 5 wt% compatibiliser did not cause a significant change in this parameter. For the ARB/10C blend, a slight increase in ultimate tensile strength was observed, amounting to approximately 3% relative to ARB, whereas for the ARB/15C sample this value was slightly lower, by approx. 3% compared with the unmodified material. This indicates that the incorporation of the compatibiliser did not cause a clear deterioration in the material’s resistance to tensile loading.

The effect of modifier content was more pronounced for tensile stress at break. ARB/10C exhibited the highest value, approx. 9% above that of ARB, and differed significantly from the other investigated blends. ARB/5C showed a value close to that of ARB, whereas ARB/15C exhibited the lowest value among the modified blends. The incorporation of the compatibiliser primarily affected the stiffness of the investigated blends, causing a significant reduction in Young’s modulus, while tensile strength remained statistically comparable to that of the unmodified ARB material. Among the modified blends, ARB/10C provided the most favourable balance of the evaluated mechanical properties and exhibited the highest tensile stress at break.

The Shore D hardness results are presented in Figure 7d. The reference material ARB exhibited the highest hardness, amounting to approx. 46 °ShD. The incorporation of the compatibiliser resulted in only a slight decrease in this parameter. For the ARB/5C blend, the hardness was approx. 45.0 °ShD, whereas for the ARB/10C and ARB/15C samples the values were similar and remained at approx. 45 °ShD.

Statistical analysis using Welch’s one-way ANOVA (OriginPro 2026) showed a significant effect of blends on Young’s modulus (F(3, 8.77) = 12.58, *p* = 0.0016), tensile strength (F(3, 7.34) = 7.65, *p* = 0.0118) and tensile stress at break (F(3, 8.08) = 99.87, *p* < 0.0001). The Games–Howell post hoc test indicated that all modified blends exhibited a significantly lower Young’s modulus than the reference ARB material, while no significant differences were observed among ARB/5C, ARB/10C and ARB/15C. For tensile strength, a significant difference was found only between ARB/10C and ARB/15C, whereas none of the modified blends differed significantly from ARB. ARB/10C exhibited significantly higher tensile stress at break than ARB, ARB/5C and ARB/15C, and a significant difference was also observed between ARB/5C and ARB/15C. No statistically significant effect of formulation on Shore D hardness was found (F(3, 19.85) = 2.14, *p* = 0.128).

The observed differences between the unmodified material and the blends containing the compatibiliser were small and did not exceed approx. 2% relative to the reference sample. This indicates that the addition of the compatibiliser did not significantly affect the hardness of the investigated polymer blends. The slight decrease in hardness may be associated with the presence of an additive with a more flexible character. However, its incorporation did not cause a marked deterioration in the material’s resistance to local surface deformation. The obtained results indicate that modification of the blend with the compatibiliser allows the hardness of the material to be maintained at a level similar to that of the ARB sample.

### 3.5. Thermal Properties Determined by Differential Scanning Calorimetry (DSC)

**Figure 8:**
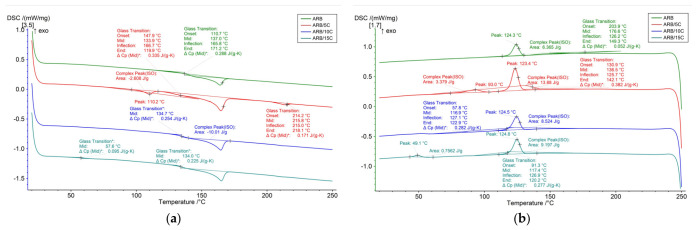
DSC thermograms of ARB blends: (**a**) heating curve and (**b**) cooling curve. * Determined according to ISO 11357-2:2020 [41].

Comparative analysis of the thermal properties of the blends was carried out using differential scanning calorimetry (DSC), and the results are presented in Figure 8a,b.

The DSC curves recorded during the second heating cycle showed a principal endothermic event in the temperature region of approximately 165–170 °C for all investigated blends. The position of this event was similar for ARB and the blends containing ENGAGE 8452, indicating that the addition of the modifier did not produce a pronounced shift in the principal melting transition.

Differences between the heating curves were observed mainly in the shape and magnitude of the endothermic effects. However, because the peak areas of the principal melting event were not determined consistently for all blends in the presented analysis, the melting enthalpies were not compared quantitatively. An additional endothermic peak at 110.2 °C was observed for ARB/5C. This effect may be associated with a transition of a minor semicrystalline component or with local compositional heterogeneity of the recycled blend.

During controlled cooling, the principal exothermic crystallisation peaks occurred within a narrow temperature range from 123.4 to 124.8 °C. The peak temperatures were 124.3 °C for ARB, 123.4 °C for ARB/5C, 124.5 °C for ARB/10C and 124.8 °C for ARB/15C. These results indicate that the addition of ENGAGE 8452 did not cause a pronounced shift in the principal crystallisation temperature.

The enthalpy of the principal crystallisation peak was 6.365 J/g for ARB, 13.88 J/g for ARB/5C, 8.524 J/g for ARB/10C and 9.197 J/g for ARB/15C. The highest value was therefore recorded for ARB/5C, followed by ARB/15C, ARB/10C and the reference ARB material. Additional exothermic events were observed at 93.0 °C for ARB/5C and at 49.1 °C for ARB/15C, with peak areas of 3.379 and 0.7562 J/g, respectively.

Overall, the principal melting and crystallisation transitions occurred within similar temperature regions for all investigated blends. The differences between the curves were mainly related to the peak areas and the presence of additional thermal events in selected samples. These observations are consistent with the heterogeneous, multicomponent character of the recycled material.

### 3.6. Microscopic Analysis

The results of the microscopic observations (SEM) of the fracture surfaces of specimens after the static tensile test are presented in Table 4.

For the reference ARB sample, the representative fracture surface exhibited a relatively heterogeneous morphology, with visible local defects, irregularities and voids, including a larger discontinuity in the central region of the examined field. These features indicate a non-uniform fracture path and local structural heterogeneity of the unmodified recycled blend. The fracture surface of ARB/5C retained a rough and irregular character but appeared more compact than that of the reference ARB sample in the representative areas examined. Regions of varied topography were visible, together with a comparatively more continuous fracture path. For ARB/10C, the examined fracture surface appeared relatively compact and continuous, with fewer clearly visible large voids and distinct discontinuities than in the reference material. Among the investigated modified blends, this sample exhibited the most uniform fracture-surface morphology in the representative micrographs. This observation is consistent with its comparatively favourable tensile stress at break, although the morphological and mechanical results should be considered as complementary observations rather than as direct evidence of a causal relationship. The fracture surface of ARB/15C also appeared relatively compact; however, local regions of more pronounced cracking and delamination were visible. In comparison with ARB/10C, the representative micrographs did not indicate a further improvement in fracture-surface continuity at the highest modifier content.

The SEM observations show that the addition of compatibiliser affected the visible fracture-surface morphology of the investigated blends. Differences were observed in surface continuity, topography and the occurrence of local voids and discontinuities. The most favourable morphological features among the modified blends were observed for ARB/10C. At the same time, rough regions and local discontinuities remained visible in all samples, which is consistent with the heterogeneous and multicomponent character of the recycled material.

## 4. Discussion

The behaviour of the investigated materials should be considered in relation to the heterogeneous composition of the automotive recycled blend. Multipoint FTIR analysis indicated the presence of, among others, polycarbonate (PC), acrylonitrile-butadiene-styrene copolymer (ABS), high-density polyethylene (HDPE) and polystyrene (PS). The coexistence of polymers differing in polarity, morphology and thermal behaviour may limit interfacial compatibility and lead to local variations in the structure and properties of the recycled material. Density-based separation reduced the complexity of the initial waste stream, but the recovered ARB fraction retained a multicomponent character, which is consistent with the challenges reported for the recycling of mixed automotive plastics [23,27,28,29,30,31,32].

The progressive reduction in blend density after the addition of ENGAGE 8452 can primarily be related to the lower density of the modifier compared with the ARB material. ENGAGE 8452 is an ethylene-octene polyolefin elastomer with a density of 0.875 g/cm^3^ and a low tensile modulus. Its incorporation therefore changes the balance between the rigid constituents of the recycled blend and the more compliant elastomeric phase. The absence of statistically significant differences in Shore D hardness indicates that this reduction in bulk density and stiffness did not produce a corresponding substantial change in resistance to local surface indentation.

The mechanical results demonstrate that the principal effect of ENGAGE 8452 was a reduction in stiffness. Welch’s ANOVA and the Games–Howell post hoc test showed that all modified blends had a significantly lower Young’s modulus than the reference ARB material. This response is consistent with the elastomeric character of the modifier and should be interpreted primarily as an increase in material compliance rather than as direct evidence of improved compatibility. At the same time, none of the modified blends differed significantly in tensile strength from ARB, indicating that the reduction in stiffness was not accompanied by a statistically significant deterioration in tensile strength relative to the reference material.

The effect of modifier content was more pronounced for tensile stress at break. ARB/10C exhibited the highest value and differed significantly from the other investigated blends. This result suggests that an intermediate modifier content provided a favourable balance between the compliant elastomeric contribution and the load-bearing capacity of the recycled blend. However, the increase in tensile stress at break cannot by itself confirm improved miscibility or interfacial adhesion. The observed response may also be influenced by changes in phase dispersion, local composition, fracture path and the distribution of defects within the heterogeneous material.

The DSC results indicate that the principal melting and crystallisation events occurred within similar temperature regions for all mixtures. Consequently, the addition of ENGAGE 8452 did not substantially shift the main thermal transitions of the semicrystalline component present in ARB. The differences in peak areas and the occurrence of additional thermal events in selected samples are consistent with variations in the contribution of minor phases and local compositional heterogeneity. In particular, the additional effects observed for ARB/5C and ARB/15C show that the thermal response of the blends cannot be described by a single homogeneous polymer phase. Because the identity and quantitative fraction of the phase responsible for the principal transitions were not established unambiguously, the enthalpy values were not converted into percentage crystallinity.

The SEM observations complement the mechanical results by showing differences in the visible fracture-surface morphology. The reference ARB material exhibited local voids, discontinuities and an irregular fracture path, whereas the modified blends generally appeared more compact in the representative areas examined. ARB/10C showed the most continuous fracture-surface morphology among the modified blends, which is consistent with its comparatively high tensile stress at break. Nevertheless, the SEM observations represent a comparative morphological assessment and should not be interpreted as direct quantitative evidence of improved miscibility or interfacial adhesion.

The combined results show that no formulation can be considered universally superior. The unmodified ARB material retained the highest stiffness and may therefore be preferable when rigidity is the principal selection criterion. The modified blends exhibited lower stiffness while maintaining tensile strength at a level statistically comparable to that of ARB. Among the modified blends, ARB/10C provided the most favourable balance under the criteria evaluated in the present study, particularly in terms of tensile stress at break, retained tensile strength, hardness, density and representative fracture-surface morphology. This assessment is limited to the investigated compositions and should not be interpreted as a universal optimum for all possible applications.

## 5. Conclusions

The conducted research demonstrates that compatibilisation is a promising method for modifying recycled polymer fractions, particularly in the context of their potential reuse in accordance with the principles of the circular economy. The incorporation of the compatibiliser enabled the ultimate tensile strength and tensile stress at break to be maintained at a level comparable to that of the unmodified material, while also affecting the structure and morphology of the blends.

Among the investigated modified blends, the most favourable balance of the evaluated properties was obtained with the addition of 10 wt% compatibiliser to the blend separated through the flotation–sedimentation separation process. The ARB/10C sample exhibited the most favourable modification effect, including the highest tensile stress at break among the investigated blends, beneficial stabilisation of mechanical properties and the most compact fracture morphology observed by SEM. These results indicate that the 10 wt% compatibiliser content may favourably affect the mechanical response and fracture-surface morphology of the investigated blend.

The developed approach enables the upgrading of the properties of a multicomponent polymer composition obtained from automotive waste. This effect can be associated with improved homogenisation of the system, reduced structural heterogeneity and more favourable interfacial interactions between the blend components. The nature of the thermal transitions observed by DSC, particularly the presence of a dominant melting effect within a similar temperature range, may indicate a more ordered character of the blend after compatibiliser modification, resembling the behaviour of a more homogeneous polymer system. Increasing the compatibiliser content to 15 wt% did not result in further improvement in the mechanical properties or morphology of the material. This may indicate that an excessive amount of the additive does not necessarily lead to more favourable effects and that its content should be optimised each time depending on the recyclate composition and the performance requirements of the final material.

The obtained results confirm that an appropriately selected amount of compatibiliser can increase the functional value of multicomponent polymer fractions derived from automotive recycling. In particular, the ARB/10C sample demonstrated potential for further use as a secondary material, supporting the possibility of reintroducing this type of fraction into the material cycle.

## Figures and Tables

**Table 1:** Symbols and composition of the analysed blends.

Symbol	Blend Composition
ARB	Automotive recycled blend—reference blend
ARB/5C	ARB blend with the addition of 5 wt% compatibiliser
ARB/10C	ARB blend with the addition of 10 wt% compatibiliser
ARB/15C	ARB blend with the addition of 15 wt% compatibiliser

**Table 2:** Mass balance and stage mass yields of the three-stage density-based separation process.

Separation Stage	Feed Mass [kg]	Light Fraction Stream [kg]	Light Fraction Stream Yield [wt%]	Heavy Fraction Stream [kg]	Heavy Fraction Stream Yield [wt%]	Mass- Balance Closure [%]
Stage 1	1.000	0.782	78.2	0.218	21.8	100.0
Stage 2	0.782	0.035	4.5	0.747	95.5	100.0
Stage 3	0.035	0.005	14.3	0.030	85.7	100.0

**Table 3:** Mechanical properties of ARB blends.

Mixture Type	Young’s Modulus [MPa]	Tensile Strength [MPa]	Tensile Stress at Break [MPa]	Shore D Hardness [°ShD]
ARB	1065 ± 90	41.8 ± 3.0	40.3 ± 2.1	45.5 ± 0.7
ARB/5C	860 ± 58	42.1 ± 1.2	41.2 ± 0.5	45.1 ± 0.5
ARB/10C	740 ± 63	42.5 ± 0.3	44.1 ± 0.3	44.9 ± 0.5
ARB/15C	765 ± 60	40.3 ± 0.9	38.4 ± 0.7	44.8 ± 0.6

**Table 4:** Comparative SEM micrographs of tensile-fracture surfaces.

Mixture Type	SEM Image	
ARB	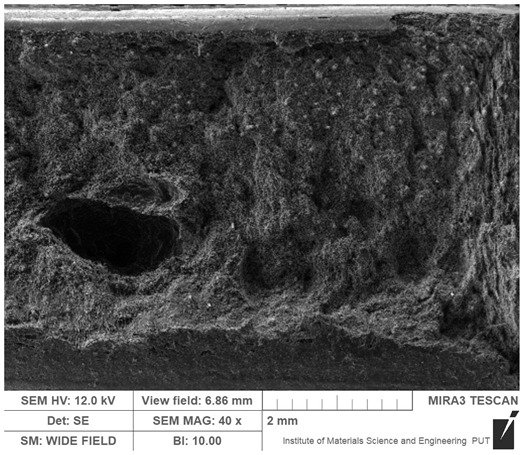	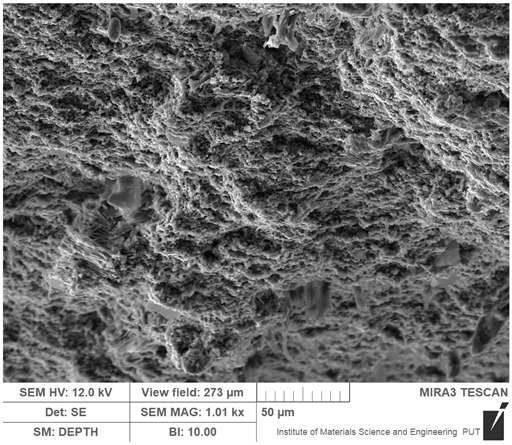
ARB/5C	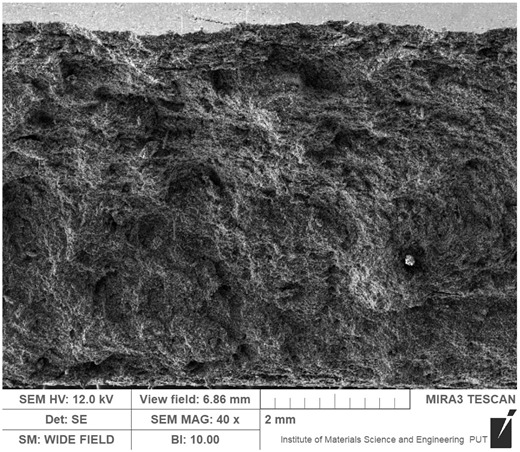	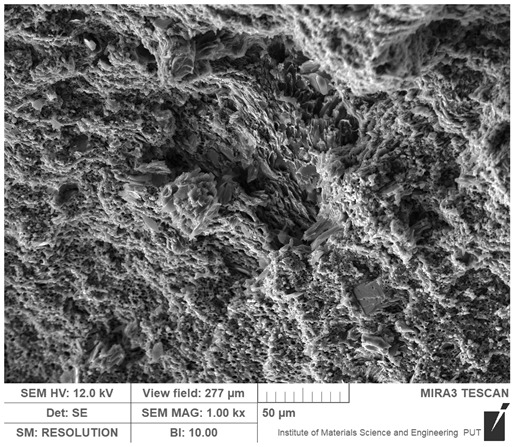
ARB/10C	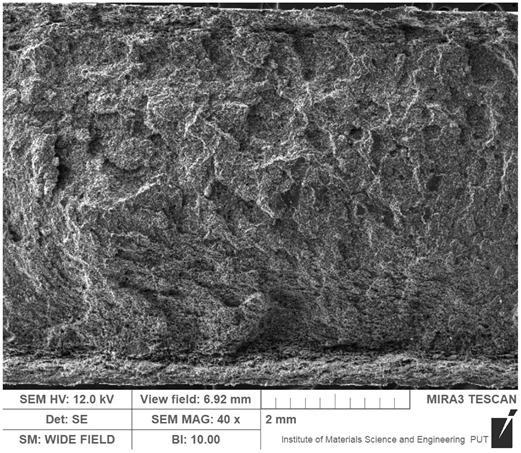	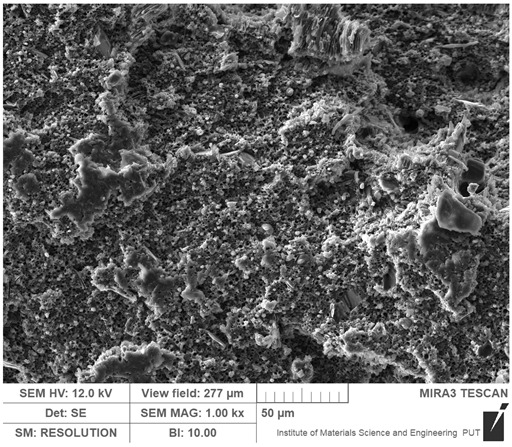
ARB/15C	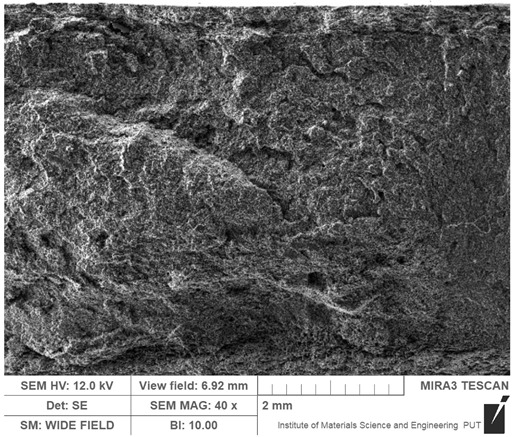	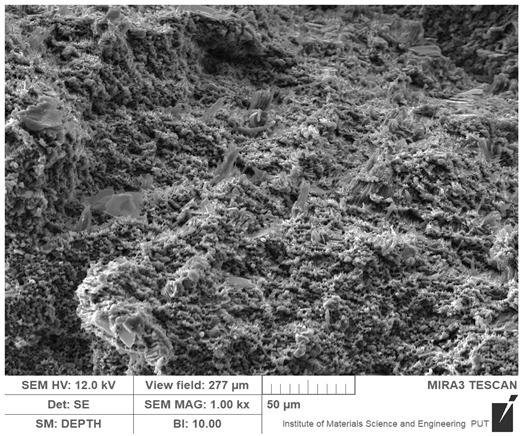

## Data Availability

The original contributions presented in this study are included in the article. Further inquiries can be directed to the corresponding author.

## Abbreviations

The following abbreviations are used in this manuscript:

ARB	Automotive recycled blend
ARB/5C	Automotive recycled blend with the addition of 5 wt% compatibiliser
ARB/10C	Automotive recycled blend with the addition of 10 wt% compatibiliser
ARB/15C	Automotive recycled blend with the addition of 15 wt% compatibiliser
SEM	Scanning electron microscope
DSC	Differential scanning calorimetry
PP/PC	Polypropylene/polycarbonate blend
FTIR	Fourier-Transform Infrared Spectroscopy
PC	Polycarbonate
ABS	Acrylonitrile-butadiene-styrene copolymer
HDPE	High-density polyethylene
PS	Polystyrene
